# A comparison of statistical methods for assessing winter wheat grain yield stability

**DOI:** 10.18699/VJ20.619

**Published:** 2020-05

**Authors:** A.F. Cheshkova, P.I. Stepochkin, A.F. Aleynikov, I.G. Grebennikova, V.I. Ponomarenko

**Affiliations:** Siberian Federal Scientific Center of Agro-BioTechnologies of the Russian Academy of Sciences, Krasnoobsk, Novosibirsk region, Russia; Siberian Research Institute of Plant Production and Breeding – Branch of the Institute of Cytology and Genetics of Siberian Branch of the Russian Academy of Sciences, Krasnoobsk, Novosibirsk region, Russia; Siberian Federal Scientific Center of Agro-BioTechnologies of the Russian Academy of Sciences, Krasnoobsk, Novosibirsk region, Russia; Siberian Federal Scientific Center of Agro-BioTechnologies of the Russian Academy of Sciences, Krasnoobsk, Novosibirsk region, Russia; Siberian Research Institute of Plant Production and Breeding – Branch of the Institute of Cytology and Genetics of Siberian Branch of the Russian Academy of Sciences, Krasnoobsk, Novosibirsk region, Russia

**Keywords:** phenotypic stability, genotype × environment interaction, plant breeding, statistical methods, winter wheat, фенотипическая стабильность, взаимодействие генотип × среда, селекция растений, статистические методы, озимая пшеница

## Abstract

The multitude of existing methods for assessing the phenotypic stability of plants makes breeders be faced with the problem of choosing an appropriate variant. The purpose of this study was to compare different methods of analyzing the genotype × environment interaction and, on their basis, assess the stability of the yield of 7 varieties of winter wheat. The article compares 17 stability statistics by applying them to data obtained from agrotechnical experiments carried in 2009–2011 for evaluating the grain yield of 7 varieties of winter common wheat of Siberian selection (Novosibirskaya 32, Novosibirskaya 40, Novosibirskaya 51, Novosibirskaya 3, Novosibirskaya 2, Obskaya winter, Omskaya 6). Analysis of variance revealed a significant ( p < 0.001) genotype × environment interaction in the experiments, which indicates a different reaction of genotypes to changes in environmental conditions. Genotypes were ranked according to the level of stability. Based on the analysis of the rank correlation matrix, the stability statistics were categorized in five groups. Recommendations were made on which group of methods to use depending on the objectives of the study. In the case when the goal of breeding research is the selection of the most biologically stable varieties with the minimum variance across a range of environments, one should use the methods of the static concept. If it is necessary to choose a genotype with a predictable reaction to changes of environmental conditions, corresponding to the calculated level or forecast, the regression approach is the most appropriate. The stability statistics generally identified Novosibirskaya 32 as the most stable variety from a biological point of view. The regression approach showed that Novosibirskaya 3 was the genotype with the smallest deviation from mean yield in all environments, while methods accessing the contribution of each genotype to the genotype × environment interaction defined Novosibirskaya 51 as the most stable variety.

## Abstract (ru)

Многообразие существующих методов оценки фенотипической стабильности растений ставит перед селекционерами проблему выбора подходящего варианта. Целью настоящего исследования было сравнение различных методов анализа взаимодействия генотип × среда и оценка на их основе стабильности ур жайности семи сортов озимой пшеницы. Проанализированы 17 статистических показателей стабильности на примере данных полевого опыта 2009–2011 гг. по оценке урожайности зерна семи сортов озимой мягкой пшеницы сибирской селекции (Новосибирская 32, Новосибирская 40, Новосибирская 51, Новосибирская 3, Новосибирская 2, Обская озимая, Омская 6) в шести вариантах сред. Дисперсионный анализ выявил значимое ( p < 0.001) взаимодействие генотип × среда в опыте, что говорит о различной реакции генотипов на изменение условий среды. Выполнено ранжирование сортов по уровню стабильности и рассчитаны корреляционные связи между параметрами стабильности. На основе анализа корреляционной матрицы рангов проведена классификация методов, разделяющая их на пять групп. Предложены рекомендации по выбору способа определения стабильности генотипов в зависимости от целей исследования. В случае, когда целью селекционных исследований является выбор наиболее стабильных в биологическом смысле сортов, обладающих наименьшей вариацией признака, независимо от меняющихся условий среды, следует использовать методы статической концепции. Если среди набора сортов необходимо выбрать генотип с предсказуемой реакцией на изменения условий среды, соответствующей расчетному уровню или прогнозу, наиболее оптимален регрессионный подход. В результате оценки методами статической концепции сорт Новосибирская 32 был определен как наиболее стабильный с биологической точки зрения. Сорт Новосибирская 3, имевший наименьшее отклонение от средней урожайности для всех сред, оказался наиболее стабильным при оценке регрессионными методами. Методы, оценивающие вклад генотипа во взаимодействие генотип × среда, определили сорт Новосибирская 51 как самый стабильный.

## Introduction

Genotype × environment (GE) interaction means that varieties react differently to changes in growing conditions. Relative ranking of the productivity of a group of varieties in different environmental conditions gives different results. Even among specific, regionally adapted breeding forms, it is difficult to choose the best genotype, due to the variability of yields in different years of observations and under different environmental conditions.

Currently, many different ways of evaluating the GE interaction are developed, which are based on statistical methods for calculating certain parameters characterizing the degree of genotype response to changing environmental conditions. Different terms are used: adaptability, homeostaticity, plasticity, stability, etc.

Becker and Leon (1988) consider that, depending on the purpose of the research, there are two main differing concepts of stability: static (biological) and dynamic (agronomic).

The static concept implies a stable genotype possesses an unchanged performance, regardless of variation of the environmental conditions. “This concept of stability is useful for traits the levels of which have to be maintained at all costs, e. g. for quality traits, for resistance against diseases, or for stress characters like winter hardiness”. The concept may include methods described by Roemer (1917, in: Becker, Leon, 1988), Francis and Kannenberg (1978), Lin and Binns (1988).

Unlike the static concept, the dynamic concept permits a predictable reaction of the genotype to changes in environmental conditions. The stable genotype, in accordance with this concept, does not deviate from the average values of this reaction. For each environment, the productivity of a stable genotype corresponds exactly to the predicted level. Among the methods of the dynamic concept used to assess stability, the following groups can be distinguished:

- methods that assess the stability of the genotype, based on the contribution to the variation of genotype × environment interaction are presented in the articles of Wricke (1962), Shukla (1972);
- a regression approach to assessing the stability of a trait is considered in the articles of Finlay and Wilkinson (1963), Eberhart and Russell (1966), Tai (1971);
- non-parametric methods based on relative data rankings were proposed by Nassar and Huehn (1987), Fox et al. (1990), Kang and Pham (1991);
- multidimensional methods that use the results of a cluster or a component analysis to assess stability were described in Fox and Rosielle (1982), Zobel et al. (1988), Lin and Binns (1991), Purchase (2000), Gauch (2006).

Comparison of different stability statistics and classification of methods was made by Lin and Binns (Lin et al., 1986), Becker and Leon (1988), Flores et al. (1998), Adugna and Labuschagne (2003), Mohammadi and Amri (2008), Mohammadi et al. (2016). Kang (1997) made a wide analysis of the GE interaction phenomenon and gave recommendations how to use this interaction for crop cultivar development.

But some methods for stability assessing suggested by Khangildin et al. (1979), Dragavtsev (Dragavtsev, Averyanova, 1983), Udachin (1990), Martynov (1990), Kilchevsky and Khotyleva (1989), were not mentioned and compared in that works.

The purpose of this study is to compare 17 different methods of analyzing the GE interaction and, on their basis, assess the stability of the yield of 7 varieties of Siberian winter wheat.

## Materials and methods

Experimental data

All analyses were performed on the grain yield data obtained from 7 varieties of winter wheat T. aestivum L.: Novosibirskaya 32, Novosibirskaya 40, Novosibirskaya 51, Novosibirskaya 3, Novosibirskaya 2, Obskaya winter, Omskaya 6. The trials were conducted at the experimental fields of the Siberian Research Institute of Plant Production and Breeding (SibNIIRS) – Branch of the Institute of Cytology and Genetics of SB RAS during three cropping seasons (2009–2011). The experiment was carried out in two variants each year: with use of mustard protection coulisses and without them. The meteorological conditions during the years of the experiments were different both in temperature and precipitation. Coulisses contributed to a greater accumulation of snow, as compared with no-coulisse plots, which allowed a long period to retain soil moisture in spring and early summer periods. Thus, contrasting growth conditions were provided, which made it possible to take into account six variants of environments for stability analysis.

The trials were evaluated using randomized complete block design with three replications. Sowing was carried out on the fallow precursor on plots of 25 m2. Seeding rate was 600 seeds/m2, sowing date was August 20–24. The grain yield was measured for each plot and then converted into tons/ha for further statistical analysis.

Statistical methods

The yield data were subjected to statistical analyses using the R (R Development Core Team, 2014) software. A model of analysis of variance (ANOVA) for v genotypes, n environments, r replications of the following form was considered:

1.

Y_ij_ = μ + d_i_ + ϵ_j_ + g_ij_ + ē_ij_

where Y_ij_ is the mean grain yield of the i-th genotype in the j-th environment (i = 1, … v; j = 1, … n); μ is the grand mean; d_i_ is the main effect of the i-th genotype; ϵ_j_ is the main effect of the j-th environment; g_ij_ is the effect of genotype × environment interaction; ē_ij_ is the GE interaction residual of the i-th genotype in the j-th environment.

Normality of model residuals was assessed by Shapiro – Wilk test (Shapiro, Wilk, 1965), and homogeneity of variance was estimated based on the criterion of Levene (1960).

The following methods were applied to experimental data (coded abbreviation was used for convenience).

1. (EV ). Roemer (1917, in: Becker, Leon, 1988) used the “environmental variance of genotypes” to determine the stability of a genotype:

**Formula:**
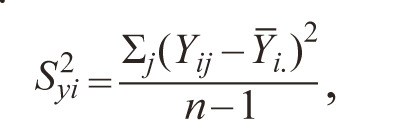
2.

where Y_ij_ is the mean value of the trait for the i-th genotype in the j-th environment (i = 1, … v; j = 1, … n); Y_i_. is the mean value of the trait for the i-th genotype in all environments. The most stable genotype has the lowest value of environmental variance. In fact S^2^_yi_ is unbiased estimation of genotype variation.

2. (CV ). Francis and Kannenberg (1978) used a combination of mean yield and standard deviation as a measure of stability. The coefficient of variation of the i-th genotype is estimated by the standard formula:

**Formula:**
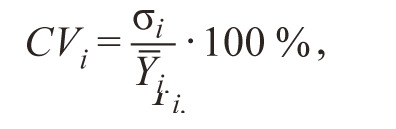
3.

where σ_i_ is the standard deviation of the trait for the i-th genotype; Y_i_. is the mean value of the trait for the i-th genotype in all environments.

Genotypes with yield above overall mean yield and CVi below overall coefficient of variation are considered more stable than the others.

3. (HOM ). Khangildin (Khangildin et al., 1979) uses the “coefficient of homeostaticity” as stability index:

**Formula:**
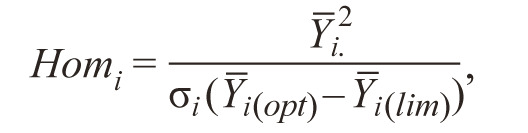
4.

where σi is the standard deviation of the trait for the i-th genotype; Y_i._, Y_i(opt)_, Y_i(lim)_ are the mean values of the trait for the i-th genotype in all environments, in the optimal and limited environment, respectively. The higher the coefficient of homeostasis is, the more stable is the genotype.

4. (Pi ). Lin and Binns (1988) proposed to use the “superiority measure” Pi as a parameter of stability:

5.

P_i_ = Σ^n^_j = 1_(Y_ij_ – M_j_)^2^/2n,

where M_j_ is the maximal value Y_ij_ for all genotypes in j-th environment. Genotypes with a lower P_i_ value are considered to be more stable.

5. (UST ). Udachin (1990) modified the method of Khangildin, proposing a new indicator – “steadiness of stability index”:

**Formula:**
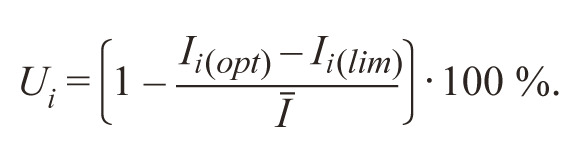
6.

Here I_i(opt)_, I_i(lim)_, I are stability indexes calculated by formulas:

**Formula:**
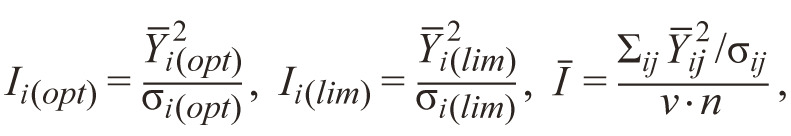
7.

where σ_ij_, σ_i(opt)_, σ_i(lim)_ are standard deviations of the trait for i-th genotype in j-th, optimal and limited environments, respectively; Y_ij_ , Y_i(opt)_, Y_i(lim)_ are average values of the trait for i-th genotype in j-th, optimal and limited environments; v is a number of genotypes, n is a number of environments. The higher this indicator is the more stable is the genotype.

6. (MAR). Martynov (1990) proposes to use a “weighted homeostacity index” for stability evaluation:

8.

H_i_ = Σ_i_a_j(k)_ (Y_ij_ – Y_.j_)/σ_j_ ,

where a_j(k)_ is the calculated weighting factor determined for the j-th environment as follows.

All environments are divided into three groups with similar conditions: favorable (Y_. j_ > Y_.._+ LSD), medium (Y_.._– LSD < < Y_.j_ < Y_.._+ LSD) and unfavorable (Y_.j_ < Y_.._– LSD). The value of LSD (least significant difference) is determined by the formula:

**Formula:**
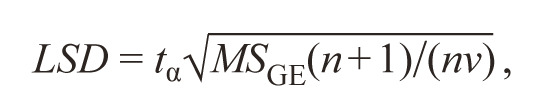
9.

where t_α_ is the value of the Student’s t-test (for selected significance level α and df = (v – 1)(n – 1)); MS_GE_ is the mean square of G × E interaction.

Let’s N_k_ (k = 1, 2, 3) – number of elements within k-th group of environments, then the weighting coefficients a_j(k)_ for each environment in k-th group are the same:

**Formula:**
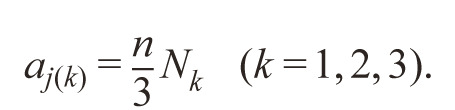
10.

The higher the H_i_ value, the more homeostatic the genotype.

7. (САС ). Kilchevsky and Khotyleva (1989) call the sum of the effects of the j-th environment and the interaction of genotype × environment as a “specific adaptive ability” of the i-th genotype in the j-th environment.

11.

CAC_ij_ = d_j_ + (vd )_ij_ = Y_.j_ – Y_.._+ (Y_ij_ – Y_i._– Y_.j_ + Y_.._ ) = Y_ij_ – Y_i.._

As a measure of the stability of the i-th genotype, they use the CAC_i_ variance:

**Formula:**
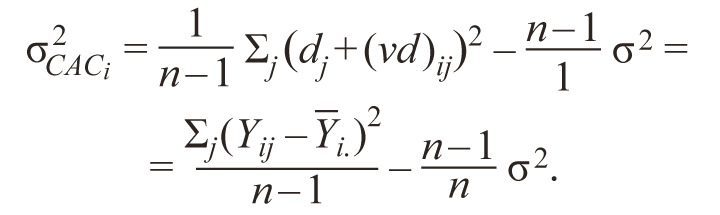
12.

8. (Wi ). Wricke (1962) proposed to use the sum of squares of environmental effects as a measure of genotype stability. This parameter is called “ecovalence” and is calculated by the formula:

13.

W_i_ = Σ_j_ (Y_ij_ – Y_i._ – Y_.j_ + Y_.._ )^2^.

The more stable genotype is, the smaller its ecovalence value will be.

9. (SIGMA). Shukla (1972) developed an unbiased estimate using “stability variance” of genotypes:

**Formula:**
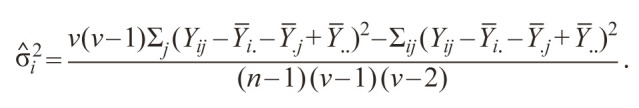
14.

10–11. (Bi, S2di). Eberhart and Russell (1966) used a regression approach to assess the stability. The following model is considered:

15.

Y_ij_ = μ_i_ + β_i_ I_j_ + δ_ij_ ,

where Y_ij_ is the variety mean of the i-th variety in the j-th environment (i = 1, … v; j = 1, … n); μ_i_ is the mean of the i-th variety over all environments; β_i_ is the regression coefficient, that measures the response of the i-th variety to varying environments; δ_ij_ is the deviation from regression of the i-th variety at the j-th environment; I_j_ is the environmental index of the j-th environment, calculated by the formula:

**Formula:**
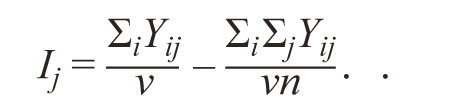
16.

The estimation of the regression coefficient b_i_ is the first parameter of genotype stability.

**Formula:**
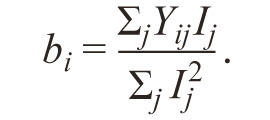
17.

The second parameter of stability is the variance of deviation from the regression line.

**Formula:**
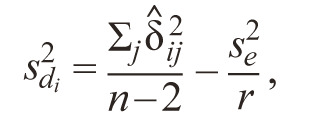
18.

where s^2^_e_ is the estimate of the pooled error; r – number of replications. The sum of the squares of deviations from the regression is calculated by the formula:

19.

Σ^j^^δ^2^_ij_ = (Σ_j_Y^2^_ij_ – (Σ_j_Y_ij_)^2^/n) – (Σ_j_Y_ij_ I_j_)^2^/Σ_j_ I^2^_j_

A variety with b^i^ = 1, s^2^_di_ = 0 are referred to stable ones. 12–13. (Alpha, Lambda). The regression approach

12–13. (Alpha, Lambda). The regression approach is also used by Tai (Tai, 1971; Tai, Young, 1972). A model of analysis of variance with random effects of the environment is considered:

20.

y^ijk^ = μ + d^i^ + ϵ^j^ + γ^k(j)^ + g^ij^ + e^ijk^ ,

where y^ijk^ (i = 1, … v, j = 1, … n, k = 1, … r) is the value of the trait of the i-th genotype in the j-th environment in the k-th replication; μ is the overall mean; d^i^ is the effect of the i-th genotype; ϵ^j^ is effect of the j-th environment; γ^k(j)^ is the effect of replicates within environments; g^ij^ is the effect of genotype × environment interaction; e^ijk^ is the residual variation due to replications.

Tai and Young (1972) proposed to determine the linear response of the genotype to the effect of the environment αi and the deviation from the linear response λ^i^. They are related to the parameters of Eberhart and Russell in the following way:

**Formula:**
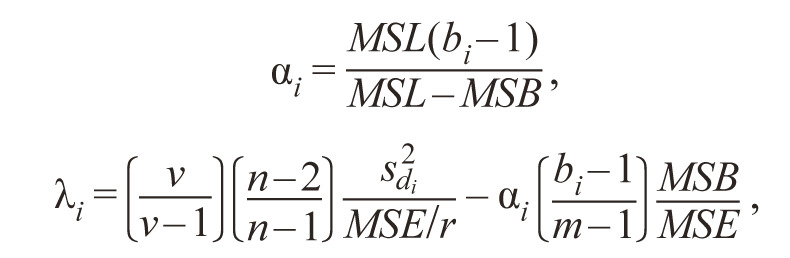
21 and 22.

where MSL, MSB, MSE are mean squares, due to environments, replicates within environments and error, respecti- vely. Tai considers that in terms of agricultural usage the preferred variety has (α = 0, λ = 1) and a mean performance which is above-average of all varieties over a series of environments.

14. (Ai ). The regression coefficient in the model of Eberhart and Russell depends on the average value of the trait. Varieties with a high average value will have a higher coefficient (scale effect). To eliminate this drawback Dragavtsev (Dragavtsev, Averyanova, 1983) proposed to use the dimensionless “coefficient of multiplicativity”:

**Formula:**
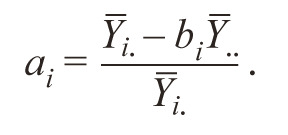
23.

15–16. (S1, S2) . Nassar and Huehn (1987) ranked genotypes in the j-th environment ( j = 1, … N ), assigning the minimum rank 1 to the genotype with the lowest corrected mean value Y^*^_ij_ = (Y^ij^ – (Y^i.^ – Y^..^)) (i = 1, … K ), and the maximum rank K to the genotype with the highest corrected mean value of the trait. A genotype is considered stable if its ranks are close for different environments. The most stable genotype has a constant rank. The following parameters are proposed as a measure of stability:

**Formula:**
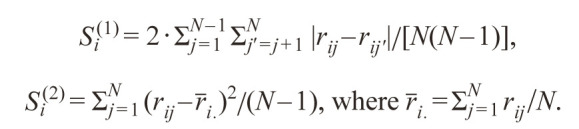
24 and 25.

For the most stable genotypes S^(1)^_i_ = 0, S^(2)^_i_ = 0.

17. (ASVI ). Zobel, Wright and Gauch (1988) suggested to use the principal component method and a graphical representation of stability based on the AMMI model (Additive Main effects and Multiplicative Interaction). The model has the form:

26.

Y^ij^ = μ + α^i^ + β^j^ + Σ^N^_k=1_ λ^k^ζ^ik^ η^jk^ + θ^ij^ ,

where Y^ij^ is the mean value of the trait of i-th genotype in j-th environment (i = 1, … v; j = 1, … n); μ is an overall mean; α^i^ is the genotype mean deviation; β^j^ is the environment mean deviation; λ^k^ is the eigenvalue of the PCA axis; ζ^ik^, η^jk^ are the genotype and environment PCA scores; N is a number of PCA axes, retained in the model; θ^ij^ is the residual.

Based on this model, Purchase (Purchase et al., 2000) proposed to use the ASV (AMMI Stability Value) parameter for ranking genotypes by stability:

**Formula:**
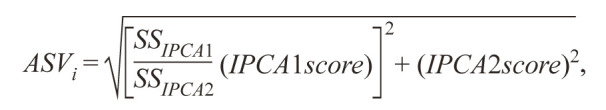
27.

where SS^IPCA1^, SS^IPCA2^ are sums of squares of the first and second principal components; IPCA1score, IPCA2score are projections of the i-th genotype on the first and second PCA axis. The smaller the value of the ASV parameter is, the more stable the genotype is considered.

18. (MeanY ). In addition, values of mean yield were calculated for each genotype using standard formulas.

For comparison of various methods for assessing stability, 17 statistical indexes described above were calculated. Then all varieties were ranked according each of the stability statistics. The ranks were assigned in such a way that the rank 1 received the most desirable variety:

- for statistics MeanY, HOM, UST, MAR, Ai, rank 1 received varieties with the highest value of the index;
- for statistics ASVI, S1, S2, Lambda, S2di, SIGMA, Wi, САС, Pi, CV, EV, rank 1 received varieties with the lowest value of the index;
- for regression coefficient Bi, rank 1 was assigned to the variety with the smallest (in modulo) coefficient’s deviation from one;
- for Alpha coefficient, rank 1 was assigned to the variety with the smallest (in modulo) coefficient’s deviation from zero.

## Results

Mean grain yield and rank of 7 genotypes across 6 environments are shown in Table 1. The fact that genotypes switch ranks from one environment to another means the presence of a crossover GE interaction.

**Table 1.**
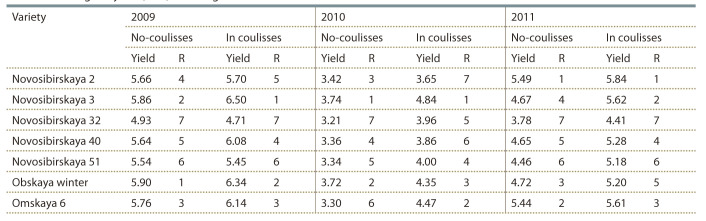
Mean grain yield (t/ha) and range of 7 winter wheat varieties tested across 6 environments

The result of analysis of variance (Table 2) shows that GE interaction is significant ( p < 0.001), which indicates a significant difference between the responses of genotypes to changes in environmental conditions. Shapiro–Wilk test showed the normality of model residuals (W = 0.99, p- value = 0.31). The Levene test proved the homogeneity of variance (F = 0.57, p-value = 0.97).

**Table 2.**
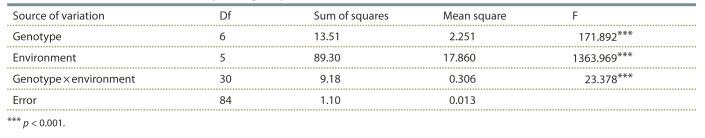
The results of the variance analysis for grain yield of 7 winter wheat varieties tested across 6 environments *** p < 0.001.

Table 3 provides the estimates of mean grain yield, 17 stability statistics and the result of ranking of the seven winter wheat varieties according these statistics.

**Table 3.**
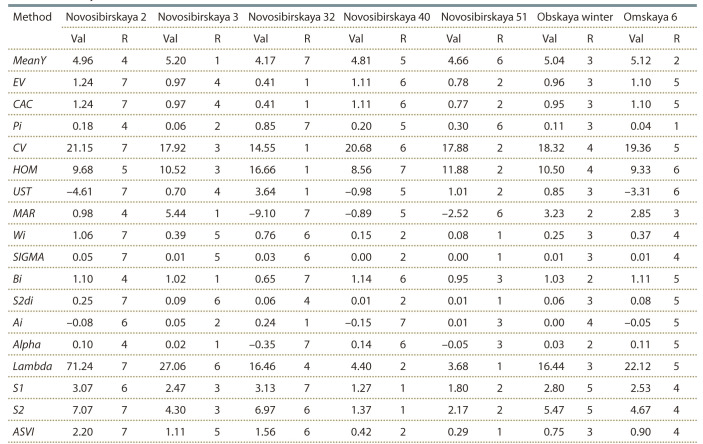
Stability statistics and ranks of 7 varieties of winter wheat tested across 6 environments Note: Refer to text for details of methods. Val is the value of the stability statistic, corresponding to the method; R is the genotype rank, corresponding to the method.

Taking mean yield as a first parameter for evaluating the genotypes, Novosibirskaya 3 gave the best mean yield while Novosibirskaya 32 had the lowest mean yield across environments. The highest variation of grain yield had Novosibirskaya 2, the lowest – Novosibirskaya 32. Genotypes Novosibirskaya 2, Novosibirskaya 3, Novosibirskaya 40, Obskaya winter, and Omskaya 6, with regression coefficients bi higher than one were able to take advantage of favorable environments, whereas Novosibirskaya 32, and Novosibirskaya 51 with b_i_ < 1 were less responsive to environmental changes. According to Shukla’s statistic, Novosibirskaya 51 provided the lowest contribution to total GE interaction, while Novosibirskaya 2 had the largest stability variance.

One can see the significant difference between methods in genotypes ranking. Such is the variety Novosibirskaya 32, which has the lowest mean yield and the lowest variance in the experiment. It is defined as the most stable one by methods focusing on the variation of the yield (EV, CAC, CV, HOM, UST, Ai), and vice versa, as the most unstable, by methods focusing on the mean value of the yield (Pi, MAR, Bi, Alpha, S1).

For further determination of the interrelationships of the methods, paired correlations of ranks defined by the stability statistics were calculated by Kendall’s method. The results are presented in Table 4 and graphically in the Figure.

**Table 4.**
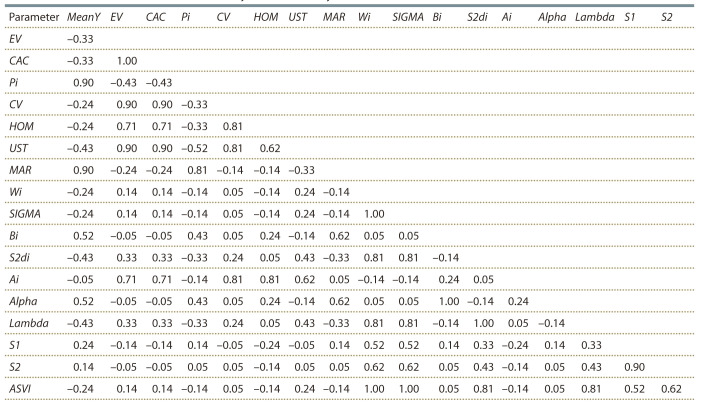
Correlations of ranks between mean yield and stability measures for 7 varieties of winter wheat Note: Refer to text for details of methods.

**Fig. 1.**
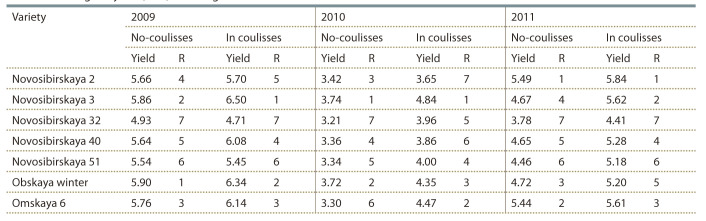
Graphic representation of the rank correlation matrix ordered by the cluster method.

High level of paired correlations was found between statistics UST, CV, CAC, HOM, Ai and EV. Pi and MAR were strongly correlated with the average yield of genotype MeanY. Another group of methods with significant paired correlations included Wi, SIGMA, S2di, Lambda, and ASVI.

Some of the considered methods were ranking genotypes identically: CAC and EV; Bi and Alpha; S2di and Lambda; Wi and SIGMA.

## Discussion

According the rank correlation matrix the considered methods can be divided into five groups determined by a high level of paired rank correlations (r > 0.8).

Group 1. Includes the regression coefficient of Eberhart and Russell b^i^ and the similar coefficient of Tai α^i^. In fact α^i^ is equal to b^i^ – 1 when the effect of replicate in environment is not considered. Becker and Leon (1988) pointed a strong correlation between b^i^ and Roemer’s environmental variance S^2^_yi_. But when we proceeded to consideration of ranks then none correlation between these parameters was found. It is explained by the fact that for environmental variance rank 1 received the variety with S^2^_yi_ closest to zero, and for regression coefficient b^i^, rank 1 was assigned to the variety with the smallest (in modulo) coefficient’s deviation from one. And so we consider b^i^ not as a measure of stability but as additional information on the average response of a genotype to advantageousness of environmental conditions.

Group 2. Includes the superiority index Pi of Lin and Binns and Martynov’s weighted homeostaticity index Hi. These statistics were strongly correlated with yield. Breeding based on these methods would favor breeding for yield, as Kang and Pham (1991) and Flores (Flores et al., 1998) found.

Group 3. Includes steadiness of stability index of Udachin Ui, coefficient of variation of Francis and Kannenberg CV^i^, variance of the specific adaptive ability of Kilchevsky and Khotyleva σ^2^_CACi_, Roemer’s environmental variance S^2^_yi_, Khangildin’s coefficient of homeostaticity Homi, Dragavtsev’s coefficient of multiplicativity a^i^. All these parameters are connected to genotype variance. The identicall genotypes ranking was received for σ^2^_CACi_ and S^2^_yi_, because the variance of specific adaptive ability of Kilchevsky and Khotyleva is nothing more than the Roemer’s environmental variance, reduced by error of experiment.

Group 4. Includes non-parametric estimates of stability of Nassar and Huehn S^(1)^_i_, S^2^_i_. Becker and Leon (1988), Adugna and Labuschagne (2003), Mohammadi and Amri (2008), reported that these statistics were highly correlated with Shukla’s stability variance ^σ^2^_i_ and with Eberhart and Russell’s s^2^_di_. But we received only medium correlation of ranks for them.

Group 5. Includes Purchase’s parameter based on AMMI model ASV^i^, ecovalence of Wricke W^i^, Shukla’s stability variance ^σ^2^_i_, Eberhart and Russell’s variance of deviation from the regression s^2^_di_, and Tai’s λ^i^ variance. The Wi and ^σ^2^_i_ parameters produced the same ranking of genotypes wich is in agreement with Kang et al. (1987). The ranks established by Wi and s^2^_di_ were highly correlated because the ecovalence and the deviation from regression are mathematically linked (Flores et al., 1998). Also, s2^2^_di_ and λ^i^ identically ranged the genotypes because they are mathematically linked. As expected, the statistics σ^2^_i_and s^2^_di_ were highly rank-correlated too. The multivariate parameter ASVi produced the same ranging of genotypes as Wi and ^σ^2^_i_ in our experiment, which is in agreement with strong correlation of these parameters demonstrated by Purchase et al. (2000).

According to Becker and Leon’s (1988) classification, the Group 3 presents methods related to the static (biological) concept of stability, and all other groups of methods refer to dynamic (agronomic) concept of stability.

Lin et al. (1986) suggested the classification of methods which contains three concepts of stability:

- Type 1: A genotype is considered to be stable if its amongenvironment variance is small.
- Type 2: A genotype is considered to be stable if its response to environments is parallel to the mean response of all genotypes in the trial.
- Type 3: A genotype is considered to be stable if the residual MS from the regression model on the environmental index is small.

According to this classification the statistics of Group 1 corresponds to Type 2 stability, those of Group 3 to Type 1, and those of Group 4 and Group 5 to Type 3 stability.

The choice of the appropriate method for evaluation the stability of genotypes depends on the objectives of the study. In the case when the goal of breeding research is the selection of the most biologically stable varieties with the minimum variance across a range of environments, the stability is defined in the sense of homeostasis, one should use the methods of the static concept from Group 3. It should be remembered that the most stable varieties in this concept may have relatively low yields. Among the varieties of winter wheat under study, the variety Novosibirskaya 32 was the most stable from the biological point of view. This variety has the smallest variance, the smallest coefficient of variation, and the greatest coefficient of homeostaticity. At the same time, this variety showed the lowest yield in the experiment.

If it is necessary to choose a genotype with a predictable reaction to changes of environmental conditions, corresponding to the calculated level or forecast, the regression approach (Group 1) is the most appropriate. The most stable will be the genotype, which has the smallest deviation from the mean yield in all environments. The variety Novosibirskaya 3 with the smallest deviation of bi from one fits this definition.

To compare the contributions of each genotype to the GE interaction, one should use the methods presented in Group 5. In these methods, the genotype that makes the least contribution to the interaction is considered the most stable. In our experiments the variety Novosibirskaya 51 was defined as the most stable from this point of view.

In the nonparametric methods of Group 4, the concept of stability is the same as in the methods of Group 5. Nonparametric methods should be used in the case when the initial data do not meet the requirements of normal distribution and homogeneity of variance. The most suitable genotype for this group was Novosibirskaya 40.

The methods from Group 2 cannot be recommended for stability evaluation, because they are strongly correlated with the mean yield and breeding based on these methods would favor breeding for yield.

## Заключение

The considered methods reflect different aspects of GE interaction. As Flores et al. (1998) mentioned: “…all the methods examined here to study the stability of genotypes are valid, although they are based on very different concepts of stability”. The breeder can choose the appropriate method depending on whether the breeding is to be based primarily on yield, primarily on stability, or simultaneously on yield and yield stability.

In our experiment the methods were applied to a small number of unique cultivars and environments. In order to find stable genotypes of winter wheat for breeding purpose farther field experiments need to be conducted.

## Conflict of interest

The authors declare no conflict of interest.
